# The role of humans in the diversification of a threatened island raptor

**DOI:** 10.1186/1471-2148-10-384

**Published:** 2010-12-13

**Authors:** Rosa Agudo, Ciro Rico, Carles Vilà, Fernando Hiraldo, José Antonio Donázar

**Affiliations:** 1 Department of Conservation Biology, Doñana Biological Station (CSIC), Av. Américo Vespucio s/n, E-41092 Seville, Spain; 2 Department of Wetland Ecology, Doñana Biological Station (CSIC), Av. Américo Vespucio s/n, E-41092 Seville, Spain; 3 Department of Integrative Ecology, Doñana Biological Station (CSIC), Av. Américo Vespucio s/n, E-41092 Seville, Spain

## Abstract

**Background:**

Anthropogenic habitat modifications have led to the extinction of many species and have favoured the expansion of others. Nonetheless, the possible role of humans as a diversifying force in vertebrate evolution has rarely been considered, especially for species with long generation times. We examine the influence that humans have had on the colonization and phenotypic and genetic differentiation of an insular population of a long-lived raptor species, the Egyptian vulture (*Neophron percnopterus*).

**Results:**

The morphological comparison between the Canarian Egyptian vultures and the main and closest population in Western Europe (Iberia) indicated that insular vultures are significantly heavier (16%) and larger (about 3%) than those from Iberia. Bayesian and standard genetic analyses also showed differentiation (*F_ST _*= 0.11, *p *< 0.01). The inference of changes in the effective size of the Canarian deme, using two likelihood-based Bayesian approaches, suggested that the establishment of this insular population took place some 2500 years ago, matching the date of human colonization. This is consistent with the lack of earlier fossils.

**Conclusions:**

Archaeological remains show that first colonizers were Berber people from northern Africa who imported goats. This new and abundant food source could have allowed vultures to colonize, expand and adapt to the island environment. Our results suggest that anthropogenic environmental change can induce diversification and that this process may take place on an ecological time scale (less than 200 generations), even in the case of a long-lived species.

## Background

The negative impact of humans on biodiversity is well known and is often referred to as 'the sixth mass extinction'. For many endangered species, humans have induced fragmentation and declines in population size that have led to strong drift in many species [e.g. [1-4]]. Species endemic to islands have paid one of the highest tolls, as shown, for instance, by the massive extinctions that followed the human colonization of the Indo-Pacific archipelagos [5]. Human colonization of islands is typically associated with habitat destruction and fragmentation, as well as with other processes such as overexploitation or introduction of exotic species and pathogens that can seriously damage species richness [6,7]. In island ecosystems above all, invasions of exotic species have been implicated as an important factor in population loss and extinction [8,9]. However, alien species may also be beneficial to some native species and act, for example, as new and abundant food resources [10,11].

The unprecedented rate of anthropogenic perturbation that has affected many regions during the last centuries may be directly or indirectly promoting changes in the selective forces acting on natural populations [12]. Consequently, human activity has become associated with evolutionary changes that occur over periods of a few hundred years, otherwise known as 'contemporary evolution' [13-15]. Several studies have reported adaptation occurring through contemporary evolution in species confronting anthropogenic environmental changes [see [16] for a review]. However, whether such anthropogenic modifications can also promote phenotypic diversification and perhaps even speciation of wild vertebrates has rarely been considered. Nonetheless, it seems unlikely that human actions would have triggered divergent evolution in vertebrate populations, especially in those species with long generation times in which evolution is expected to proceed at a relatively slower pace than species with short generation times [17,18].

In this study, we examine the role of humans in the origin of the phenotypic and genetic divergence of the Canarian population of Egyptian vulture (*Neophron percnopterus*). The Egyptian vulture is a long-lived trans-Saharan migratory raptor that is globally threatened [19]. This vulture is one of the few raptors that has colonized islands far from continental mainlands and it has established sedentary insular populations such as the one on the Canarian archipelago. Our results demonstrate that the arrival of humans in the Canary Islands enabled the establishment of Egyptian vultures and their subsequent demographic explosion and differentiation.

## Methods

### Study species and populations

The Egyptian vulture (*Neophron percnopterus*) is a long-lived medium-sized scavenger bird of prey that is widely distributed throughout the circum-Mediterranean region and sub-Saharan Africa, as well as in the Middle East, Central Asia and India. Insular populations occur in the Atlantic Ocean and the Mediterranean and Arabian Seas, although many of these are now extinct [20-22]. Despite its wide distribution, this vulture is globally threatened and, due to recent population declines, it is presently classified as 'Endangered' on the IUCN Red List [19]. The main causes for its decline are high mortality of adult individuals caused by poisoning, collisions with wind power turbines and electric lines, electrocution, loss of suitable habitat and food shortage due to human disturbance [19].

At present, the bulk of the European breeding population is restricted to the Iberian Peninsula (Iberia) with approximately 1500 breeding pairs [19]. In the Canarian archipelago, it was very abundant in the past [23], but has disappeared from five of the seven islands in recent decades [24]. Most of the Canarian population is found on Fuerteventura (the southeasternmost island) where intensive monitoring over the last 12 years has revealed the presence, in average, of 30 breeding territories/year (SD = 6.4). In addition, between two and four breeding pairs are usually observed every year during the breeding season on the closest island, Lanzarote, which is located less than 10 km from Fuerteventura [[25,26], authors' unpublished data]. However, these individuals are normally seen in Fuerteventura during the rest of the year, where they have been captured and banded. Other birds from Fuerteventura are occasionally observed in Lanzarote but they spend most of the time in Fuerteventura where the bulk of the population remains and more food is available.

This study is based on samples from Iberia (n = 143) and from Fuerteventura (n = 242) which includes approximately the 85% of the current insular population. The total population was estimated at about 200 birds in 2009 (author's unpublished data).

### Field procedures and morphological analyses

Birds were captured, ringed and sampled between 1995 and 2000 in Iberia and between 1998 and 2007 in Fuerteventura. Fledglings were captured in their nests and adult and immature birds were captured with cannon nets at supplementary feeding points in every sampled area. Birds were aged on the basis of plumage features [20]. All individuals were weighed (in g) and standard body measurements were taken (in mm): length of wing chord, bill, culmen, seventh primary, tail and tarsus. To test for differences in morphological traits between the two studied populations, first we conducted a principal component analysis (PCA) of all the measured variables. Then, we performed a MANOVA test including one variable from each axis [weight (g), wing chord (mm) and bill length (mm)] and age and sex as covariates (see results for details).

### Genetic analyses

#### Genetic diversity, population differentiation and detection of migrants

DNA was extracted from blood samples from a random subset of the individuals sampled in Iberia (n = 96) and all samples available from the Canarian islands (n = 242), using a standard phenol-chloroform extraction [27]. Individual sex was determined in the lab by amplifying a fragment of the sex chromosomes Z and W using a polymerase chain reaction (PCR) with primers 2550F and 2718R [28]. The presence and size of amplification products was assessed by agarose electrophoreses. Genetic diversity was assessed using five autosomal microsatellite loci developed for the Bearded vulture (*Gypaetus barbatus*) [29] and 17 species-specific microsatellites [30]. We used GENALEX version 6 [31] to calculate parameters of genetic variability and the differences between the two populations was tested using Wilcoxon sign-rank tests.

Population structure was measured by *F*_ST _[32] that was tested for significance by performing 10,000 permutations with the programme GENETIX[33]. Since this measure of differentiation/fixation is limited to some extent by the diversity of the markers [34], we also calculated the standardised measure of genetic differentiation of Hedrick [34] (G'_ST_) using SMOGD[35]. Additionally we used the programme STRUCTURE v.2.2 [36], which employs a Bayesian clustering method to infer the most likely number of populations (*K*) assuming no *a priori *structure. First, we investigated the most likely *K *running five independent simulations of *K *= 1-3. All simulations were run using default parameters in the admixture model and with correlated allele frequencies. Each run included 100,000 iterations of burn-in, followed by 500,000 iterations used for parameter estimation. The most likely value of *K *was chosen using the Δ*K *statistic, based on the rate of change between successive *K *values, as proposed by Evanno et al [37]. Then, non-residents or potential migrant individuals in each of the proposed clusters were identified using posterior probabilities calculated for each individual in STRUCTURE using the "usepopinfo" option.

In order to confirm the suggested migrant individuals by STRUCTURE, and detect other potential migrants from unsampled populations, we performed an assignment test implemented in GENECLASS 2.0[38,39]. This program uses likelihood-based statistics in combination with resampling methods. Given that we may have not sampled all potential source populations, we used two different likelihood-based test statistics. First we estimated *Lh*, the likelihood of finding a given individual in the population in which it was sampled. This is the most appropriate statistic to use when all potential source populations have not been sampled [38,39]. We also used *Lh/Lmax*, the ratio of *Lh *to the greatest likelihood among all sampled populations [38], which has greater power and is most informative when all source populations have been sampled. We employed the Bayesian criterion of Rannala & Mountain [39] and the resampling method of Paetkau *et al*. [38] to determine the critical value of the test statistic (*Lh *or *Lh/Lmax*) beyond which individuals were assumed to be migrants. We selected an alpha level of 0.01 to determine critical values, as simulated data have shown this level to represent an appropriate balance between stringency and power [38].

Finally, to assess if the differentiation observed between the two populations could be explained without any gene flow, we used EASYPOP[40]. This program allows simulating multilocus population datasets under a large array of conditions. We simulated two populations diverging as a result of genetic drift and without any gene flow (we performed 100 replicates). We assumed monogamy, 1000 males and 1000 females for the continental source population and 40 females and 40 males for the insular deme. Each individual in the simulation was characterized by 22 unlinked loci with a maximum of 15 alleles per locus (values as those in our dataset) with average mutation rate of 5 × 10^-4 ^and 95% single step mutations [41].

#### Demographic history

In order to estimate the date of population establishment in the Canarian archipelago, we investigated historical changes in the effective size of the Canarian population using two likelihood-based Bayesian methods. The Beaumont method [42] implemented in the programme MSVAR 0.4 assumes that a stable population of size *N1 *started to decrease or increase *ta *generations ago toward the current population size *N0*. The change in population size is assumed to be either linear or exponential and mutations are assumed to occur following a step-wise mutation model (SMM). Based on these assumptions and using a Bayesian coalescent-based Markov chain Monte Carlo (MCMC) approach, it is possible to estimate the posterior probability distribution of three demographic parameters scaled by the current effective population size (*N0*): *r = N0/N1 *(rate of population size change), *tf = ta/N0 *(time since the population size change started) and θ = 2*N0*μ, where μ is the mutation rate. Since we are testing founder and bottleneck effects, the simulations were run under the exponential growth model. Given that this method does not allow a straight forward calculation of the time of population change (*ta*), this was calculated from *tf *after independently determining the current effective population size (*N0*) using the linkage disequilibrium method implemented in the program N_E_ESTIMATOR [43]. For the date calculations we estimated the species generation time (average age at which the females give birth to offspring; [44]) to be around 13 years, using the data from the long-term monitoring of marked individuals [[45,46], authors' unpublished data).

We validated the results from the Beaumont method by obtaining another estimate of the time of population change with another method (the hierarchical model) developed by Storz and Beaumont [47]. This method is implemented in MSVAR 1.3 and quantifies the effective population sizes *N0 *and *N1 *and the time *T *(in generations) since the population size change started. It assumes an exponential change in population size and prior distributions for *N0, N1*, *T *and θ are assumed to be lognormal. Briefly, this method differs from the original model [42] in three main aspects: 1) in the original model, multiple loci are accommodated by estimating posterior densities of the parameters for each locus separately and then taking the product of the independent densities. In the second method, posterior densities for both models are estimated using all loci in the same MCMC simulation. 2) In the original model the inferred parameters are scaled by current population size (*N0*) but in the second model the parameters are inferred separately using priors, following the approach of Tavaré et al [48] and Wilson and Balding [49]. 3) The original model is based on the assumption that all parameters other than mutation rate were identical across loci. However, in the hierarchical model parameters are free to vary from one locus to the next (for more details see [47])

For both methods we used wide uninformative priors and we performed multiple runs to evaluate the stability of the estimates. The total number of iterations was larger than 2 × 10^8 ^and thinning intervals ranged from 2 × 10^4 ^to 5 × 10^4^. First 10% of the updates were discarded to avoid biases in parameter estimation due to the starting conditions as recommended by the author [42]. The remaining data were used to obtain the median (50%), and the lower (10%) and upper (90%) quantiles of the posterior distributions of the parameters. Consistency in the shape of the posterior distributions from the individual runs was examined to evaluate the convergence of the output values.

## Results

### Morphologic and genetic differentiation

**Figure 1:**
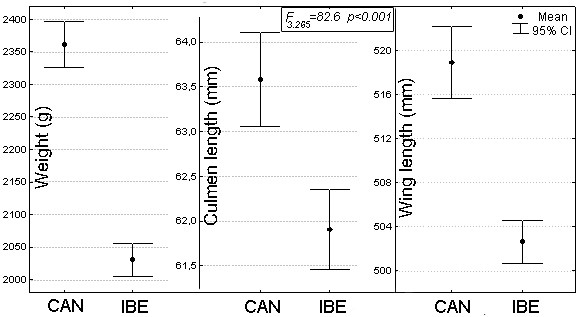
**Mean and 95% confidence interval for three morphological traits in two vulture populations**. (CA: Canary Islands, n = 242; IBE: Iberian Peninsula, n = 143). Results from the MANOVA test are shown.

The PCA extracted three main components that accounted for 77.7% of the initial variance. The first component (48.9% of the variance) included two variables with positive loadings: bill length (loading = 0.88) and culmen length (loading = 0.87). The second component (15.7%) clustered measurements of wing chord (loading = 0.77) and primary length (loading = 0.92). Finally, positive values in the third component (13.1%) were only related to weight (loading = 0.90). Therefore, we performed the MANOVA test with one variable from each axis [weight (g), wing chord (mm) and bill length (mm)]. This analysis indicated an overall significant difference between populations (Wilks' Lambda = 0.52, *F_3.265 _*= 82.6, *p *< 0.001, Partial Eta Squared = 0.48) without effects of age and sex (p > 0.05). Results showed that Canarian Egyptian vultures are significantly heavier (16%) and larger (about 3% for both wing chord and bill length) than those from Iberia (Figure 1).

Genetic analyses indicated that the Canarian population had lower genetic diversity, with an average expected heterozygosity of 0.442 and an allele richness of 2.44, than the peninsular population, which had estimates of 0.562 and 2.98 respectively (Z = 2.46, p = 0.01 and Z = 2.13, p = 0.03). Genetic differentiation between the two populations showed that the insular and the Iberian populations were moderately genetically differentiated (*F_ST _*= 0.11, *p *< 0.01). The standardised genetic differentiation measure G'_ST _provided a value of 0.168 indicating an important differentiation. Most microsatellite loci appeared to conform to the stepwise mutation model; four loci had at least one allele that had a length change different than the repeat unit (one base pair difference). This small portion of loci have unlikely affected our results.

**Figure 2:**
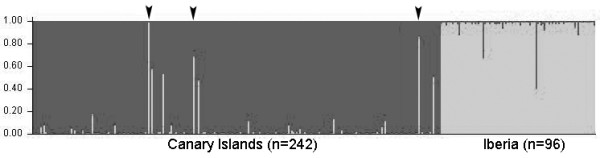
**Clustering analysis in STRUCTURE without considering information about population of origin (*k *= 2)**. Individuals are represented as vertical bars, where the amount of each colour indicates the proportion of each inferred cluster. Sampled populations are indicated (Canary Islands, N = 242; Iberia, N = 96). Those canarian individuals that were significantly identified as migrants (or of migrant ancestry) when using the "usepopinfo" option of STRUCTURE (see results, figure not shown), are indicated in this figure with black arrows.

Calculation of the statistic Δ*K *[37] from the STRUCTURE runs indicated that two (*K *= 2 (Δ*K *= 457.3; Figure 2) was the most likely number of clusters (Iberia and the Canaries, averaged of 5 runs for *Ln P(X|K) *= (-14808.16) for *K *= 1 and (-13746.54) for *K *= 2). All runs at *K *= 2 produced identical clustering solutions with similar values of cluster membership *q *for all individuals. Almost all individuals (except for some possible migrants and their descendents, see below) from Canary Islands were assigned to their population with *q *> 0.85, and vultures from Iberia were assigned to a single cluster with *q *> 0.84.

### Detection of migrants

Using sampling location as prior information for STRUCTURE (*K *= 2), we identified two individuals from the Canary Islands (06P and 035) as potential migrants (probability of membership to the Iberian population: *q *= 0.96 and 0.823, respectively), and one individual (0R6) as potentially having migrant ancestry (*q *= 0.48). None of the peninsular individuals seemed to have originated from the islands. Assignment tests performed with GENECLASS were concordant and also identified these individuals as migrants. We did not detect any other potential migrant individual that corresponded to an unsampled population (Table 1).

**Table 1:** Results of the migrant detection analysis from STRUCTURE and from GENECLASS from which all individuals with probabilities of assignment to their population of origin <0.05 for either one of the two statistics (Lh, Lh/Lmax), are shown. Populations are: IBE: Iberia and CAN: Canary Islands.

		STRUCTURE	GENECLASS		
ID (ring)	Origin	*q* (with pop. information)	migrants F_0 _[-log (*Lh*)]	Prob. pop. origin *Lh*/(*Lh*/*Lmax*)	assigned population [-log (L)]
		*K *= 2 (IBE|CAN)			
06P	CAN	0.956|0.00	27.04	0.00/0.00	IBE (19.29)
035	CAN	0.823|0.00	19.39	0.017/0.018	IBE (19.35)
0R6	CAN	0.479|0.474|	17.74	0.037/0.028	IBE (17.37)

### Simulation of population differentiation

**Figure 3:**
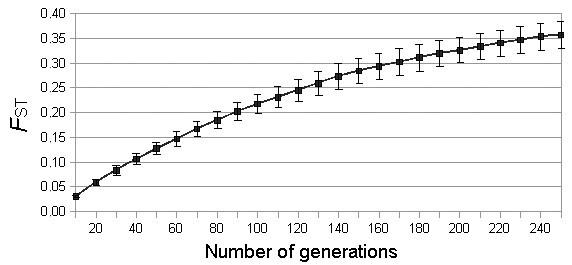
**Increasing differentiation (*F*_ST_) with time between two populations diverging by drift alone, without gene flow**. Averaged ***F*_ST _**values and standard deviation deriving from 100 replicates simulated in EASYPOP mimicking the Iberian and Canarian populations of Egyptian vultures (see text).

In order to investigate if the differentiation observed between the two populations could be compatible with complete isolation since the colonization of the islands, we simulated how this differentiation could proceed in the absence of gene flow using EASYPOP. The simulations indicated that the two populations would need about 40 generations (over 500 years) in complete isolation to reach the *F*_ST _values observed between the Canarian and the Iberian populations (0.11) (Figure 3). Given that the origin of the insular population is much older than 500 years (see below) this result indicates that occasional immigration may have contributed to limit the population differentiation.

### Estimate of size and date of population change

**Figure 4:**
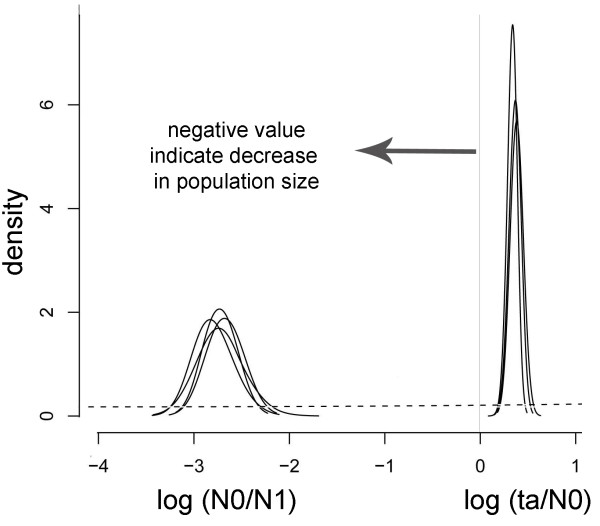
**Population size change**. Posterior distributions of the demographic parameters on a logarithmic scale obtained with the Beaumont (1999) method: (*r*) = (*N0*/*N1*) represents the ratio of present (*N0*) to past (*N1*) population size; (*tf*) = (*ta*/*N0*) represents the ratio between the time in generations (*ta*) of the population change and the present population size (*N0*). Every solid line corresponds to a different run and the prior distribution is shown for comparison (flat dashed line).

Results from the Beaumont method suggested a strong decrease in the Canarian Egyptian vulture population size. The posterior density distribution for *log(N0/N1) *is shown in Figure 4 together with the flat prior (dotted line) for comparison, and indicates a reduction in effective population size of about three orders of magnitude (*log(N0/N1)~-*3). The posterior density of *log(ta/N0) *indicates an average value of 0.39 (10^th^-90^th ^percentiles = 0.29-0.48) (Figure 4). Time in generations (*ta*) for the population collapse was calculated by using the estimate of the current effective population size for the Canarian population calculated by the linkage disequilibrium method. This method yielded an estimation of 38.8 effective individuals (ranged from 36.1 to 41.7), which closely matched the current number of successful breeding birds (mean number of breeding pairs during the last 8 years = 35, mean productivity = 0.54; unpublished data from the authors). Based on this estimated effective population size, we calculated that a past population bottleneck took place around 191 generations or 2,461 years ago (median value, 10^th^-90^th ^percentiles = 2,056-2,892) and the pre-bottleneck effective population size (*N1*) was of 21,442 individuals (10^th^-90^th ^percentiles = 10,905-38,780).

**Figure 5:**
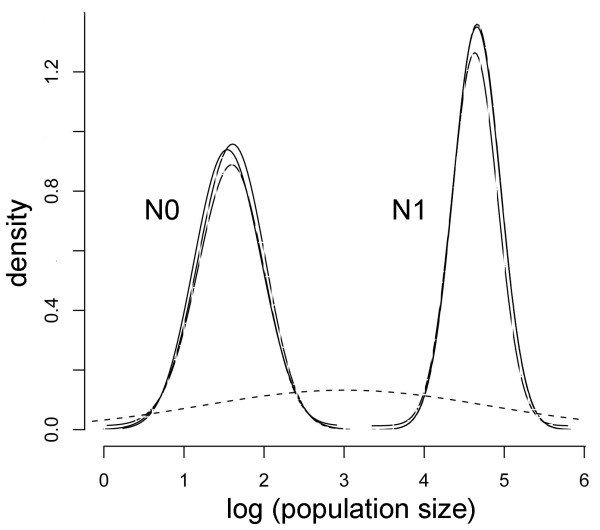
**Ancestral and present population sizes**. Posterior distributions on a logarithmic scale for past (*N1*) and current (*N0*) effective population sizes from the Storz and Beaumont (2002) method. Every solid line corresponds to a different run and prior distribution is shown (dashed line).

**Figure 6:**
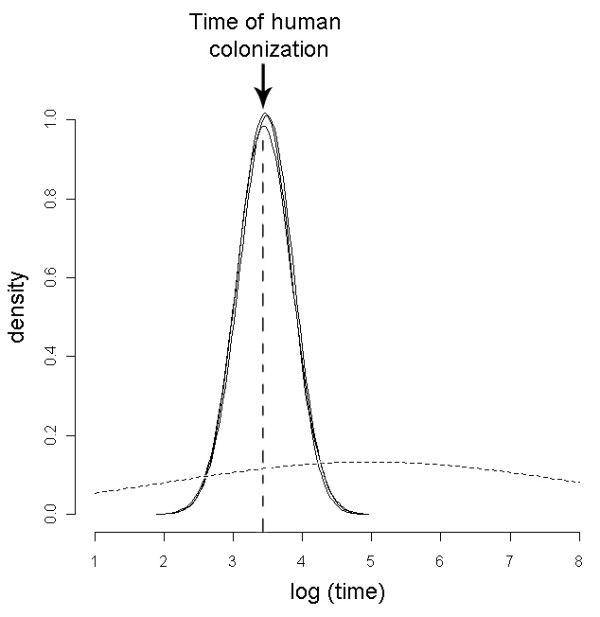
**Time of the population bottleneck (founding event)**. Posterior distribution on a logarithmic scale of the date (in years) for the Canarian population founding event, obtained with the Storz and Beaumont (2002) method. Every solid line corresponds to a different run. The prior is shown as a dashed line with median 100,000 years ago and the arrow corresponds to the date of human colonization as indicated by the archaeological record (about 2,500 years ago).

Results from the Storz and Beaumont methodsupported these findings and showed no overlap between the posterior distributions for *log(N0) *and *log(N1)*. The posterior densities were very different from the priors used (Figure 5, dashed line) and indicated a strong signature of a population bottleneck. These results suggested a past effective population size *(N1) *of 45,842 (10th-90th percentiles = 19,159-109,591), a current effective population size *(N0) *of 38 (10th-90th percentiles = 11-122) (Figure 5) and a genetic bottleneck 2,924 years ago (median value; 10th-90th percentiles = 880-9,130) (Figure 6). These results corroborate the estimates obtained with the previous approach for the current effective size and the time of the bottleneck. Although the divergence was larger between the estimates of *N1 *when it was calculated with the Storz and Beaumont method, both approaches suggest a very large effective ancestral population.

## Discussion

The Bayesian analysis of the historical demography of the Canarian Egyptian vulture population revealed the existence of a bottleneck approximately 2500 years ago and an ancestral effective population of tens of thousands of individuals. It is unrealistic to assume that this estimate represents the former Canarian population for which, moreover, no fossil evidence exists. Even though the fossil chronology of the Quaternary Period is well preserved and birds are one of the best represented groups, especially in Fuerteventura [50], the only remains of Egyptian vultures appear to be modern [51]. This would suggest that the species was rare or absent from the islands until recently. The absence of large terrestrial mammals could well have precluded the successful colonization of the islands by large scavengers. Food resources available to vultures in the Canary Islands before the arrival of domestic animals were scarce and variable since they would have consisted only of the remains of seabirds and sea mammals, or of rodents [50,52]. Even though shoreline carrion is a valuable resource for some vulture populations [53], it is probably not sufficient for maintaining a stable reproductive population in islands as small as those of the Canarian archipelago.

It is thus more likely that the estimated effective population size of tens of thousands of birds corresponds to the ancestral source population from which the founders of the Canarian population originated. Consequently, the date of the bottleneck suggested by the genetic data would correspond to the date the insular population was established, which closely matches the arrival of the human colonizers of these islands about 2500 years ago [50]. Archaeological remains show that these first inhabitants were Berber people from northern Africa, who imported and maintained herds of goats (*Capra hircus*). Subsequent chronicles dating from the European conquest in the fifteenth century describe large numbers of goats, with more than 60,000 on Fuerteventura (1659 km^2^) [50]. Hence, the arrival of humans and subsequent livestock could have provided sufficient food resources to enable colonization by Egyptian vultures. In historical accounts from the sixteenth to twentieth centuries, these birds are described as very abundant and dependent on domestic animals [23,50,54].

The introduction of this new and abundant food source by humans could have allowed not only the colonization by these vultures, but also their demographic expansion and their putative adaptation to the new island environment. The phenotypic differences observed between the Canarian Egyptian vultures and their potential source population (Iberia) may be due to drift, resulting from the isolation and small effective population size in the islands. However, some morphological and ecological changes observed in the insular vultures are compatible with various characteristic features associated with insularity: Canarian birds are sedentary [55], exhibit tendency to gigantism [56] and are tamer [57].

Our genetic analyses reveal clear divergence and support the current classification of the insular deme as a separate subspecies (*N. p. majorensis*) [58]. This differentiation indicates that admixture between the Iberian and Canarian populations may be rare. However, the finding of two immigrant Iberian birds and one individual of admixed ancestry on the islands substantiates the fact that, like many other trans-Saharan European species [59], Egyptian vultures occasionally reach the archipelago. The migratory route of these vultures crosses the Western Sahara desert and can, on occasions, pass along the West African coast, only 95 kilometres away from the Canary Islands and a crossable distance for this species [[20], unpublished data from the authors]. These observations suggest that, although Iberian Egyptian vultures could have been able to regularly reach this archipelago, they were unable to establish a stable population until the arrival of humans and goats.

This unique Canarian Egyptian vulture population has suffered a precipitous decline during the second half of the twentieth century caused by mortality due to human persecution [25]. However, food availability has never been a concern for the conservation of the species in the islands [25,60]. Goat-raising is still the most important economic activity in Fuerteventura and goat carcasses are still this species' primary source of food [11]. It is paradoxical that while human activities are behind the origin of this divergent lineage, other human activities are contributing to its demise.

## Conclusions

The bottleneck associated with the colonization of the Canarian archipelago (followed by demographic expansion), together with the presumably different selective pressures of a new environment, may have promoted diversification in this species, which has occurred over less than 200 generations. Therefore, our results show that anthropogenic environmental changes can induce vertebrate diversification and that this process can take place on an ecological time scale, even in the case of long-lived species.

## Authors' contributions

RA participated in the sample collection, carried out the molecular genetic lab work and the genetic analyses, participated in the design of the study and drafted the manuscript. CR participated in the lab work and helped to draft the manuscript. CV and FH helped to draft the manuscript and analyses. JAD supervised the design of the study, participated in the sample collection, performed the morphological studies and helped to draft the manuscript. All authors read and approved the final manuscript.
